# Experimental Therapy of Paracoccidioidomycosis Using P10-Primed Monocyte-Derived Dendritic Cells Isolated From Infected Mice

**DOI:** 10.3389/fmicb.2019.01727

**Published:** 2019-07-31

**Authors:** Leandro B. R. Silva, Cleison L. Taira, Lucas S. Dias, Ana C. O. Souza, Joshua D. Nosanchuk, Luiz R. Travassos, Carlos P. Taborda

**Affiliations:** ^1^USP-LIM53, Laboratory of Medical Mycology, Institute of Tropical Medicine, University of São Paulo, São Paulo, Brazil; ^2^Department of Microbiology, Institute of Biomedical Sciences, University of São Paulo, São Paulo, Brazil; ^3^Departments of Medicine (Division of Infectious Diseases) and Microbiology and Immunology, Albert Einstein College of Medicine, New York, NY, United States; ^4^Department of Microbiology, Immunology and Parasitology, Federal University of São Paulo, São Paulo, Brazil

**Keywords:** paracoccidioidomycosis, dendritic cells, peptide P10, vaccine, monocyte-derived dendritic cells

## Abstract

Paracoccidioidomycosis (PCM) is an endemic mycosis in Latin American caused by the thermodimorphic fungi of the genus *Paracoccidioides* spp. Notably, a Th1 immune response is required to control PCM. In this context, dendritic cells (DCs) seem to be essential players in capture, processing and presentation of *Paracoccidioides* antigens to naïve T cells and their further activation. We have previously demonstrated that differentiated DCs from bone marrow cells, pulsed with the immunoprotective peptide 10 (P10), effectively control experimental PCM immunocompetent and immunosuppressed mice. However, this procedure may not be infeasible or it is limited for the therapy of human patients. Therefore, we have sought a less invasive but equally effective approach that would better mimics the autologous transplant of DC in a human patient. Here, we isolated and generated monocyte differentiated dendritic cells (MoDCs) from infected mice, pulsed them with P-10, and used them in the therapy of PCM in syngeneic mice. Similar to the results using BMDCs, the P10-pulsed MoDCs stimulated the proliferation of CD4^+^ T lymphocytes, induced a mixed production of Th_1_/Th_2_ cytokines and decreased the fungal burden in murine lungs in the setting of PCM. The process of differentiating MoDCs derived from an infected host, and subsequently used for therapy of PCM is much simpler than that for obtaining BMDCs, and represents a more reasonable approach to treat patients infected with *Paracoccidioides*. The results presented suggest that P10-primed MoDC may be a promising strategy to combat complicated PCM as well as to significantly shorten the lengthy requirements for treatment of patients with this fungal disease.

## Introduction

Paracoccidioidomycosis (PCM) is a systemic granulomatous disease caused by the thermally dirmophic fungus of the Paracoccidioides genus and it is one of the most important systemic diseases in the Latin America, where it occurs from Mexico to Argentina (reviewed by Taborda et al., 2015). In Brazil, between 1996 and 2006, approximately 1,853 (∼51.2%) of 3,583 confirmed deaths due to systemic mycoses were caused by PCM (Prado et al., 2009). A Th_2_–type immune response, characterized by high levels of IL-4, IL-5, and IL-10 cytokines as well as increased levels of IgG4 and IgE subclass antibodies, is associated with the most severe clinical form of this mycosis, the acute-subacute PCM (de Castro et al., 2013). In contrast, the chronic form of the disease similarly results in the down-modulation of Th_1_ type responses, but polarization toward Th_2_ reactivity may not occur (de Castro et al., 2013).

Peptide P10, mapped as being the immunodominant antigenic region of the glycoprotein 43 (Gp43) of the fungus *Paracoccidioides brasiliensis*, comprises a sequence of 15 amino acids that contains specific epitopes for T-CD4^+^ cells. Mice immunized with P10 had a 200-fold lower fungal burden in their lungs as compared to non-immunized mice (Taborda et al., 1998), and had higher amounts of IFN-γ and IL-12. We have also demonstrated the protective effect of P10-primed dendritic cells (DCs) differentiated from bone marrow (BMDCs) against murine PCM (Magalhães et al., 2012; Thind et al., 2015; Silva et al., 2017). DCs play important regulatory roles during the innate and adaptive immunity in *P. brasiliensis* infection. DCs are strategically injected in a different anatomical sites and they migrate through non-lymphoid peripheral tissues, continuously examining the environment for foreign compounds and abnormal cells, including invasive microorganisms, to capture, prosecute and present antigens to activate T cells (Shortman and Naik, 2007). Hence, they are considered “professional APCs” (Banchereau and Steinman, 1998; Janeway and Medzhitov, 2002; Romani et al., 2004). During infection, peripheral DCs are activated by interaction with microorganisms or inflammatory mediators, which are responsible for stimulating the increased expression of MHC and co-stimulatory molecules, such as CD80, CD86, and CD40 (Shortman and Liu, 2002; Fonteneau et al., 2003). DCs are mostly derived from bone marrow hematopoietic cells (BMDCs). However, inflammation or infection can promote the generation of DCs from monocyte infiltrates (Serbina et al., 2003). These DCs have been termed “inflammatory DCs” (iDCs) or “monocyte-derived DCs” (MoDCs) (Mildner et al., 2013; Segura and Amigorena, 2013), which share a similar expression of MHC-II, CD11b, and CD11c with BMDCs, but differentially express CD64, the Fc-gamma receptor 1 (FcgRI) (Tamoutounour et al., 2012; Plantinga et al., 2013).

Different fungal pathogens induce variable activation of DCs, resulting in the expansion of distinct T-helper cells (Romani, 2011). The differentiation of Th_1_, Th_2_ or Th_17_ immunity hardly depends on the innate immune receptor engaged in the recognition of the fungal pathogen, and the subsequent production of cytokines by DCs. The production of IL-12 is associated with Th_1_ differentiation, while IL-23, together with TGF-β and IL-6, intensifies the Th_17_ phenotype. The differentiation and expansion of regulatory T cells (T_regs_), which are responsible for controlling excessive inflammation, is facilitated by the predominant production of IL-10 and TGF-β (Bellocchio et al., 2004; Netea et al., 2006; Robinson et al., 2009; Romani, 2011).

Here, we evaluated the use of P10-stimulated MoDCs, as a therapy for murine PCM. In this therapeutic approach, we generated MoDCs from mice infected with *P. brasiliensis*, pulsed them with P10, and injected them back into syngeneic mice with an established infection with *P. brasiliensis*. This model most closely mimics the theoretical therapy using autologous transplantation of DC for a human patient with PCM. Using this approach, we have shown that the injection of P10-primed MoDC stimulates the proliferation of T CD4^+^ and T CD8^+^ lymphocytes and the production of cytokines that are known to favor the control of PCM. In addition, we showed that vaccination with P10-primed MoDCs significantly reduced lung fungal burden during murine PCM. These results reinforce P10-primed MoDCs vaccination as an effective candidate for PCM therapy and further indicate that studies with human patients should be actively pursued.

## Materials and Methods

### Animal Use and Ethics Statement

Male BALB/c mice aged 6 to 8 weeks were obtained from the Animal Facility at the University of São Paulo (USP)’s School of Medicine in pathogen-free conditions with *ad libitum* access to chow and water. The National Council of Ethics with Animals (CONCEA) guidelines were strictly followed in the course of this project and the protocol used in this study was approved by the Ethics Committee on Animal Experiments of USP (authorization number 189/14).

### Fungus and Inoculum Preparation

The standard, virulent isolate *P. brasiliensis* 18 (Pb18) was maintained on Sabouraud agar for 7 days at 37°C. To prepare the inoculum used in the experiments, Pb18 was harvested from the tubes and washed three times with phosphate buffered saline (PBS, pH 7.2). Thereafter, large and agglutinated yeast cells were separated by decanting, and the small isolated yeast cells were collected and counted by hemocytometer. The yeast cells used for intratracheal (i.t.) infection were >95% viable as determined by Trypan blue staining.

### Intratracheal Infection

Infection by the i.t. route was performed in BALB/c mice anesthetized intraperitoneally (i.p.) using a 200 μl solution of 80 mg/kg of Ketamine and 10 mg/kg of Xylazine (both from the União Química Farmacêutica, Brazil). After receipt of anesthesia, a small longitudinal incision was made in the neck, exposing the trachea, and 50 μl of PBS containing 3 × 10^5^ Pb18 were injected. This highly reproducible infection model results in a chronic pulmonary infection (Taborda et al., 1998; Magalhães et al., 2012).

### Peptide Synthesis and Purification

Peptide 2.0 (Chantilly, VA, United States) produced the P10 peptide at a purity of ≥98% as determined by HPLC and mass spectrometry. Endotoxin (lipopolysaccharide) concentration was <0.1 EU/mL, measured by the Limulus amebocyte Lysate QCL-1000 (Lonza, Walkersville, MD, United States).

### Bone Marrow Dendritic Cell Differentiation From *P. brasiliensis* Infected Mice

The bone marrow derived dendritic cells (BMDC) used in this study were obtained according to an established protocol (Inaba et al., 1992). Briefly, after 30 days of infection with Pb18, mice were euthanized and their femurs and tibias were flushed with RPMI medium to release bone marrow cells. These cells were then washed in RPMI, counted with a hemocytometer and seeded at 10^7^ cells/plate/10 ml in 90 mm × 15 mm Petri dishes with RPMI supplemented with IL-4 (15 ng/ml), rGM-CSF (30 ng/ml) (both from Invitrogen), 20 μg/ml gentamicin (Gibco BRL Life Technologies, NY, United States), and 10% fetal calf serum (Gibco). On the third and fifth day, 10 ml of fresh medium was added to the plate with growth factors. On the seventh day, differentiated BMDCs were obtained.

### Monocyte-Derived DCs From Infected Mice With *P. brasiliensis*

Cardiac puncture was performed in anesthetized mice 30 days after infection with Pb18 and the blood was diluted in saline 0.9% (NaCl) at a 1:1 ratio. After dilution, the blood was transferred to Falcon tubes containing ficoll at 1: 2 in diluted blood and centrifuged for 20 min at 2,000 rpm. After centrifugation, the mononuclear cells located at the interface between the ficoll and the supernatant were collected and transferred to a new Falcon tube where the volume was filled with saline and then centrifuged again for 10 min at 1,500 rpm. The pellet was collected and suspended in RPMI medium. The cells were counted in an hemocytometer and plated onto 12-well tissue culture plates at 5 × 10^6^ cells/well and incubated with 5% CO_2_ at 37°C. After 2 h, non-adherent cells (predominantly lymphocytes) present in the supernatant were removed. The remaining adherent cells (predominantly monocytes) were cultured in RPMI with 20 μg/ml gentamicin (Gibco), IL-4 (35 ng/ml) and recombinant GM-CSF (50 ng/ml) (both from Invitrogen), and 10% FBS (Gibco). Fresh medium was added on the third and fifth day of culture, and the differentiated MoDCs were obtained on the eighth day.

### Enrichment of DCs

After differentiation, BMDCs and MoDCs were washed with MACs buffer consisting of PBS with 1% bovine serum fetal (Gibco) and 2mM EDTA (VWR International, Leuven, Belgium). Negative cell depletion was carried out using CD3, CD4, CD19, and F4/80 MicroBeads and LD columns according to the manufacturer’s instructions (Miltenyi Biotec). Briefly, 10^7^ cells the cells were suspended in 70 μl of MACS buffer and CD3, CD4, CD19, and F4/80 MicroBeads (10 μl of each) were added and incubated for 15 min at 4°C. After incubation and washing, the cells were suspended in 1 ml of buffer up to 1.25 × 10^8^ cells. LD columns were prepared by rinsing with 2 ml of buffer, and 10^6^–10^8^ labeled cells (out of 10^7^ - 5 × 10^8^ total cells) were applied onto one column. The columns were washed with 2 ml × 2 ml of buffer and total effluent containing the unlabeled cell fraction was collected. The cells were stored at 4°C prior to use.

### Presentation of P10 by BMDCs and MoDCs

For characterizing the antigen presentation of P10 to host effector cells, 10^6^ cells/ml of BMDC or MoDC were seeded into 24-well plates in RPMI medium with or without P10 (255 μM) for 2 h in CO_2_ at 37°C. Next, the cells were collected, counted in a hemocytometer, and 3 × 10^5^ cells in 100 μl were injected subcutaneously in mice that were previously infected with Pb18 30 days earlier. Controls groups received subcutaneous injections with PBS.

### MoDCs and BMDC Phenotypes Identified by Flow Cytometer

BMDCs and MoDCs differentiated or not in the presence of P10, were washed with FACS buffer and incubated with antibodies for immunophenotype differentiation (Table 1) or activation (Table 2). The gate strategy used for immunophenotyping is provided in the Supplementary Figure S1 (BMDCs) and Supplementary Figure S2 (MoDCs). After the addition of the antibodies, the cells were incubated for 30 min at 4°C in the dark. The non-ligand antibodies were washed in FACS buffer and the DCs were homogenized in FACS buffer and fixed with 1% paraformaldehyde. Sample acquisition was performed on a flow cytometer (FACSFortessa – Becton Dickinson, Palo Alto, CA, United States). Sample analysis was performed using the FlowJo 10 software (TreeStar). For each population, the mean fluorescence intensity (MFI) and the percentage of positive cells for the activation markers were evaluated.

**TABLE 1:** Monoclonal antibodies used for immunophenotyping of dendritic cells after differentiation from bone marrow or circulating monocytes.

**Antibodies**	**Fluorochrome**	**Clone**
CD11c	BV711	N418
CD8a	FITC	53–6.7
MHC-II	APC-Cy7	M5/114.15.2
Ly6C	BV605	AL-21
CD115	PE	AFS98
Viability	EF450	

**TABLE 2:** Monoclonal antibodies used for determining activation profiles of dendritic cells differentiated from bone marrow or circulating monocytes pulsed or not with P10.

**Antibodies**	**Fluorochrome**	**Clone**
CD11c	BV711	N418
MHC-II	APC-Cy7	M5/114.15.2
CD115	PE	AFS98
Ly6C	BV605	AL-21
CD8a	FITC	53–6.7
CD80	PerCP-Cy5.5	GL-1
CD86	APC	15-10A1
Viability	EF450	

### Co-culture and Lymphoproliferation Using Carboxyfluorescein Succinimidyl Ester (CFSE)

After differentiation and enrichment, MoDCs and BMDCs were plated onto 96-well plates (4 × 10^4^ cells/well). Splenocytes from mice after 30 days of infection were stained with 5 μM CFSE in sterile PBS with 2% of FBS at room temperature in the dark for 15 min. After incubation, the labeling was stopped with equal volume of frozen FBS. After washes in RPMI 1640, the cells were suspended in complete RPMI medium (1% non-essential amino acids, 1% sodium pyruvate, 10% Fetal Bovine Serum, 2 mM L-glutamine, 50 μM b-mercaptoethanol, 50 μg/ml ciprofloxacin). The cells were counted in a Neubauer’s chamber and 2 × 10^5^ cells seeded together with the MoDCs and BMDCs (5 splenocytes: 1 DC) in 96-well plate. The plate was incubated in CO_2_ at 37°C for 96 h. After that, the supernatant culture was removed for cytokine analysis, the cells were stained with a cocktail of fluorescent antibodies (Table 3), and the proliferation of T cells was evaluated by flow cytometry (Supplementary Figure S3).

**TABLE 3:** Activation profiles of the BMDC and MoDC CD8 subsets pulsed with P10.

	**CD8- Subtypes**			**CD8+ Subtypes**		
		
	**MHC-II**	**CD80**	**CD86**	**MHC-II**	**CD80**	**CD86**
BMDC	0	0	0	0	0	0
MoDC	0	0	0	0	0	0
BMDC P10	+	0	+	+	+	+
MoDC P10	+	0	+	+	0	+

*Bone Marrow-derived Dendritic Cells (BMDC) and Monocyte-Derived Dendritic Cells (MoDC) were obtained after *in vitro* differentiation in the presence of GM-CSF and IL-4. The activation profiles of BMDCs and MoDCs were determined by expression of MHC-II, CD80, and CD86 molecules quantified by flow cytometry. A significant increase in (*p* < 0.05) the Median Fluorescence Intensity (MFI) after the cells were pulsed with P10 is represented by (+), while no differences is represented by (0). Data shown are representative of two independent experiments, analyzed by one-way ANOVA followed by Tukey’s post-test.*

### Immunotherapy of Murine PCM With P10-Differentiated DC

After differentiation, enrichment and stimulation with P10, BMDCs, and MoDCs (3 × 10^5^ cells per) were subcutaneously injected in the mice abdomen. The treatment was performed only once, 30 days after infection. BMDCs and MoDCs without P10 priming were used as control treatments.

### Fungal Burden in the Lungs of Infected Mice

Mice were euthanized 60 days after infection (i.e., 30 days after therapy with DCs or injection with PBS) and their lungs were removed. To analyze the fungal burden by colony forming units (CFU), sections of each lung were removed, weighed and homogenized in PBS. Aliquots of 100 μl were plated onto Brain Heart Infusion (BHI) agar supplemented with 5% spent supernatant from *P. brasiliensis* Pb192 cultures, 10 IU/ml streptomycin-penicillin (Cultilab, Brazil), 4% fetal bovine serum (Gibco), and 500 mg/ml cycloheximide (Sigma, St. Louis, MO, United States) (Moscardi-Bacchi et al., 1994). CFUs were determined after 10 days of incubation at 37°C.

### Cytokine Detection by Cytometric Bead Array (CBA)

Cytokines from the isolated and macerated lungs were measured with BD CBA Mouse Th_1_/Th_2_/Th_17_ Cytokine Kit and BD CBA Mouse Inflammation kit according to the manufacturer’s instructions (BD Bioscience, San Jose, CA, United States). The Th_1_/Th_2_/Th_17_ kit was used for the simultaneous detection of mouse IL-2, IL-4, IL-6, IFN-γ, TNF-α, IL-17A, and IL-10 in a single sample. The Inflammation kit was used for the simultaneous detection of the mouse, IL-6, IL-10, monocyte chemoattractant protein-1 (MCP-1), IFN-γ, TNF-α, and IL-12p70. The kits were used according with the company instructions. Samples were measured on the BD FACS Fortessa Flow Cytometer and analyzed by FCAP Array 3 Software (BD Bioscience). The theoretical limits of detection for the kit Th_1_/Th_2_/Th_17_ were 0.1 pg/mL for IL-2, 0.03 pg/mL for IL-4, 1.4 pg/mL for IL-6, 0.5 pg/mL for IFN-γ, 0.9 pg/mL for TNF, 0.8 pg/mL for IL-17A, and 16.8 pg/mL for IL-10 and for the inflammation kit were 5 pg/mL for IL-6, 17.5 pg/mL for IL-10, 52.7 pg/mL for MCP-1, 2.5 pg/mL for IFN-y, 7.3 pg/mL for TNF, 10.7 pg/mL for IL-12p70.

### Histological Analysis

Lung sections were fixed in 10% buffered formalin, embedded in paraffin and sectioned. Sections (5 μm) were stained with Gomori–Grocott method and hematoxylin/coisin (HE).

### Statistical Analysis

*In vivo* experiments were performed twice independently, with experimental groups composed of 6 animals each. *In vitro* experiments were performed in triplicate for each condition and repeated 3 times. GraphPad Prism 7 software (San Diego, CA, United States) was used to run the statistical analysis. The results were expressed as mean values and standard deviations (SD) of the indicated values. One-way ANOVA with Tukey’s post-test was employed for non-parametric data. p values of ≤0.05 were used to indicate statistical significance.

## Results

### *In vitro* Differentiation of BMDCs and MoDCs in the Presence of P10

**FIGURE 1:**
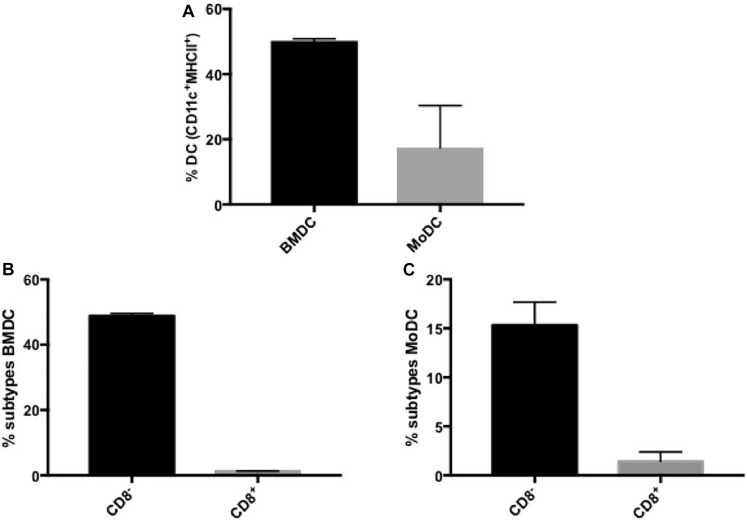
Dendritic cells derived from bone marrow or monocytes have differential expression of CD8. Bone Marrow-derived Dendritic Cells (BMDC) and Monocyte-Derived Dendritic Cells (MoDC) were obtained after *in vitro* differentiation in the presence of GM-CSF and IL-4, and evaluated for CD11c and MHC-II expression by flow cytometry **(A)**. In addition, BMDCs **(B)** and MoDCs **(C)** were classified according to the expression of CD8. Data are representative of two independent experiments.

We had previously shown that BMDCs derived from healthy mice and primed with P10 were effective in the therapy of both immunocompetent (Magalhães et al., 2012) and immunosuppressed (Silva et al., 2017) murine models of PCM. In order to develop a more clinically achievable approach that would better mimic the therapy of PCM using autologous transplant of DCs in human patients, we decided to investigate the efficacy of MoDCs primed with P10 in a murine model. First, we compared the efficiency of the *in vitro* differentiation of BMDCs and MoDCs, originally obtained from infected mice, regarding the expression of CD11c and MHC-II markers (Supplementary Figures S1, S2). The efficiency of the *in vitro* differentiation of DC from bone marrow was higher than from monocytes, since ∼50% of BMDCs were CD11c^+^MHC-II^+^, whereas only ∼20% of MoDCs were CD11c^+^MHC-II^+^ (Figure 1A). Then, we analyzed two DCs subpopulations according to the expression of CD8 (Supplementary Figures S1, S2). We found that BMDCs (Figure 1B) and MoDCs (Figure 1C) are predominantly CD8^–^. Nevertheless, the stimulation with P10 increased the expression of CD86 and MHC-II in both CD8^–^ BMDCs and MoDCs, while it increased the CD80 expression only in CD8^+^ BMDCs (Table 3 and Supplementary Figures S1, S2). Interestingly, microscopy and flow cytometry analysis demonstrated that the MoDCs obtained from either healthy or infected mice bear a distinct morphology in comparison with BMDCs, with MoDCs being more heterogeneous in size and complexity (data not shown).

### *Ex vivo* Lymphoproliferation Assay and Cytokine Production Induced by P10-Primed DCs

**FIGURE 2:**
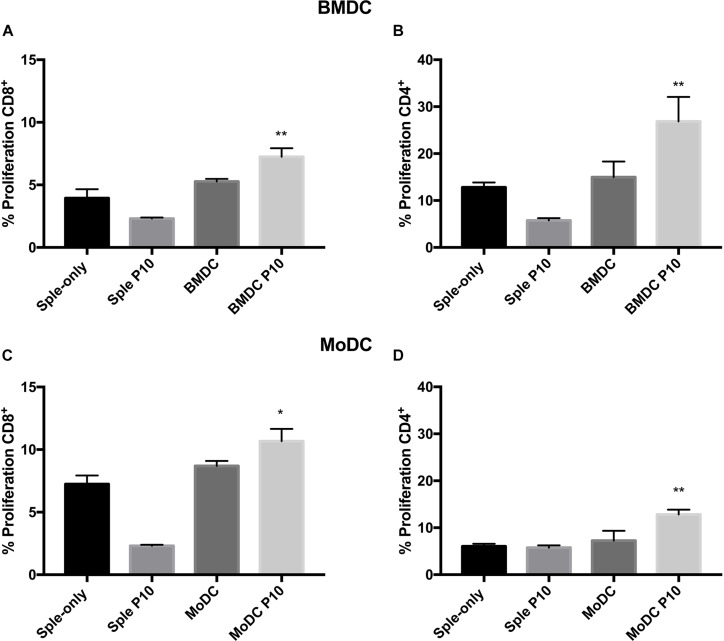
Proliferation of CD4 and CD8 T lymphocytes. Splenocytes from mice infected with *Paracoccidioides brasiliensis* were incubated with BMDCs **(A,B)** or MoDCs **(C,D)** previously pulsed with P10, and after 96 h the proliferation was analyzed by flow cytometry using the CFSE dilution method. Splenocytes cultivated in the absence (splenocytes) or presence of P10 (sple+P10) or co-incubated unprimed DC (BMDC or MoDC) were used as controls. ^*^Statistical significance between the splenocyte+DC and splenocyte+DCP10 groups. Data shown are representative of two independent experiments, analyzed by one-way ANOVA followed by Tukey’s post-test, where ^*^p < 0.05 and ^∗∗^*p* < 0.01.

After determining the influence of P10 stimulation in the differentiation of BMDCs and MoDCs, we evaluated the ability of these purified DC cell types to induce T-cell responses through co-culture with splenocytes derived from Pb infected mice (Supplementary Figure S3). The DC’s skill to present antigens to T cells was indirectly evaluated using an *ex vivo* cell proliferation assay, where splenocytes were stained with CFSE and were co-cultured with DCs. The lymphoproliferation was determined by CFSE fluorescence dilution measured by flow cytometry. In this system, both BMDCs and MoDCs primed with P10 were able to induce the proliferation of CD4^+^ and CD8^+^ T cells in comparison with DCs that were not primed (Figure 2). However, this proliferation has some remarkable characteristics in both types of DCs. While P10-primed BMDCs (Figures 2A,B) induce proliferation of CD4^+^ T more than CD8^+^ T cells, MoDCs (Figures 2C,D) induce similar proliferation levels of both T cell subsets. As the T cells were obtained from infected animals, the proliferation assay shows that P10-specific memory T cells are generated during the infection.

**FIGURE 3:**
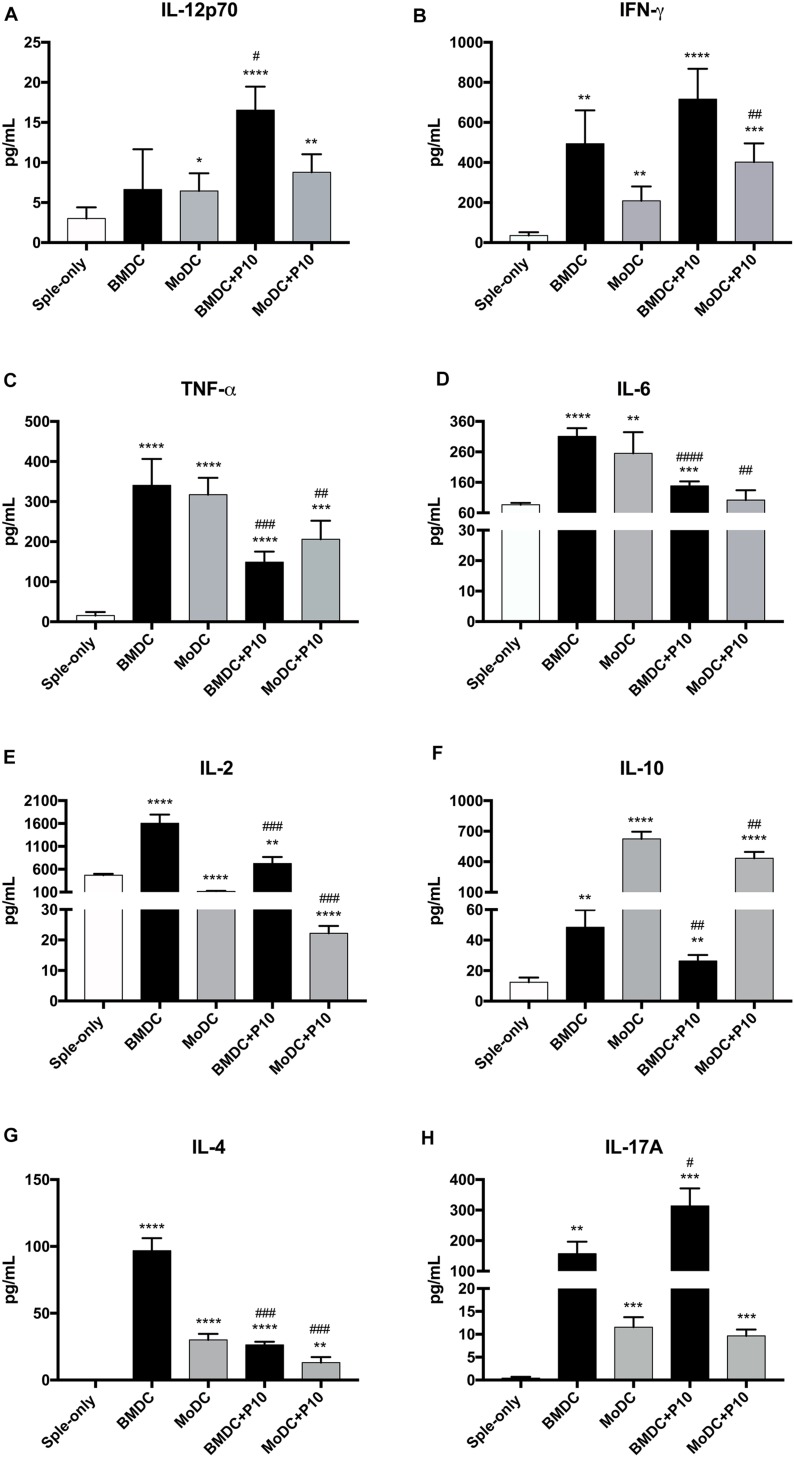
Cytokine profile in the supernatant of co-culture of splenocytes and BMDC or MoDC pulsed or not with P10. Splenocytes from mice infected with *P. brasiliensis* were co-cultivated with BMDC or MoDC pulsed (BMDC+P10 or MoDC+P10) or not (BMDC or MoDC) with P10, and after 96 h the measurement of cytokines in the supernatant was analyzed by CBA. Splenocytes cultivated alone (Sple-only) were used as controls. The data are representative of two independent experiments. Data were analyzed by one-way ANOVA followed by Tukey’s post-test, where ^*^p < 0.05, ^∗∗^*p* < 0.01, ^∗∗∗^*p* < 0.001, or ^*⁣*⁣**^p < 0.0001 in comparison to Sple-only (control culture with DC) and #*p* < 0.05, ##*p* < 0.01, ###*p* < 0.001, or ####*p* < 0.0001 for comparison of respective BMDC or MoDC groups pulsed or not with P10. **(A–H)** correspond respectively to cytokines, IL-12p70, IFN-γ, TNF-α, IL-6, IL-2, IL-10, IL-4, IL-17A.

We also determined the cytokine concentrations in the co-culture supernatants obtained after 96 h of incubation with BMDCs or MoDCs (Figure 3) that were pulsed or not with P10. Alterations in the production of the majority of cytokines tested were observed in the co-culture supernatants in comparison to the splenocyte cultures where DCs were absent. Splenocytes cultures with BMDCs or MoDCs that were not primed with P10 induced an increase in IFN-γ, TNF-α, IL-6, IL-10, IL-17A, and IL-4 levels in the co-culture supernatants cytokines, whereas IL-12 p70 increase and IL-2 decreased level, in the co-culture supernatant with MoDCs, when compared with splenocyte group. In the splenocytes culture with BMDCs or MoDCs pulsed with P10, we observed increase in IL-12 p70, IFN-γ, TNF-α, IL-6, IL-10, IL-17A, and IL-4 in the culture supernatants, whereas IL-2 decreased in the co-culture supernatant MoDC pulsed with P10, when compared with culture supernatant splenocyte group. However, comparing the supernatant of co-culture BMDCs and MoDCs not pulsed with P10 with the supernatant of co-culture BMDCs or MoDCs pulsed with P10, we observed increase of IFN-γ level in co-culture with MoDCs pulsed with P10 and in the co-culture of BMDC pulsed with P10, IL-12 p70, and IL-17A increased as well. The cytokines TNF-α, IL-6, IL-2, IL-10, and IL-4 decreased level in the supernatant co-culture BMDCs or MoDCs pulsed with P10 when compared with the group BMDCs or MoDCs not pulsed with P10.

### PCM Therapy With BMDCs or MoDCs Primed or Not With P10

**FIGURE 4:**
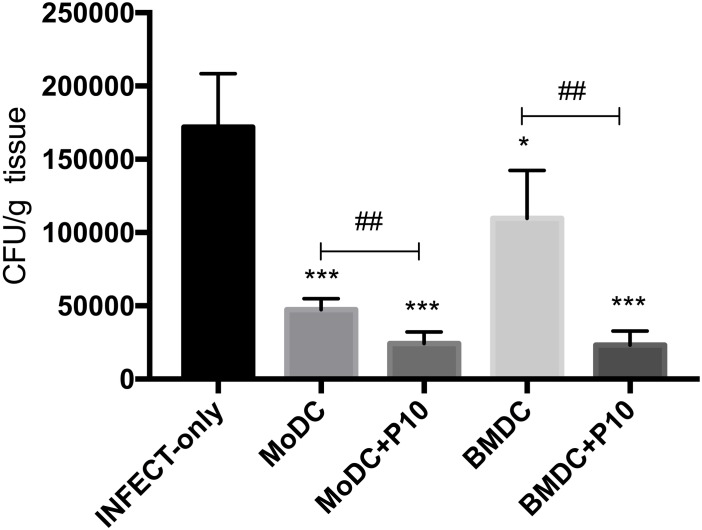
Therapeutic delivery of BMDC or MoDC pulsed or not with P10 significantly reduces pulmonary fungal burden. Mice (*n* = 6) were infected intratracheally with *P. brasiliensis*, and after 30 days the animals were treated once with MoDC or BMDC pulsed or not with P10 by subcutaneous route. The animals were euthanized 30 days after the DC treatment and the pulmonary fungal load was evaluated by CFU counts in BHI plates. Untreated mice (INFECT-only) were used as control. Data shown are representative of three independent experiments, analyzed by one-way ANOVA followed by Tukey’s post-test, where ^∗∗∗^*p* < 0.001 in comparison to INFECT-only and ##*p* < 0.01 for comparison of respective DC pulsed or not with P10. ^*^p < 0.05.

Our data demonstrated that both BMDCs and MoDCs derived from infected mice and differentiated in the presence of P10 induced increased proliferation of CD4^+^ and CD8^+^ T cells, and they stimulated the production of cytokines *ex vivo*. These results indicated that P10-differentiated DCs stimulated the development of Th_1_ responses, which are protective against PCM. Next, we evaluated the ability of P10-differentiated DC derived from infected mice to modify PCM in mice. The pulmonary fungal burden of mice infected with *P. brasiliensis* was significantly reduced by administration of DCs differentiated in the presence or not of P10 (Figure 4). BMDCs and MODCs that were not primed with P10 were able to significantly reduce the fungal burden in the lungs by ∼40 and ∼70%, respectively, compared to the burdens in infected and untreated mice. However, P10-primed MoDCs and BMDCs were even more effective, with both modalities resulting in ∼85% reduction in fungal burden relative to infected controls.

**FIGURE 5:**
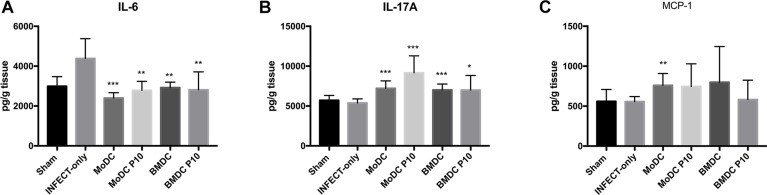
Cytokines measurement by CBA in the lung after therapeutic administration of BMDC or MoDC pulsed or not with P10. Mice (*n* = 6) were infected intratracheally with *P. brasiliensis*, and after 30 days the animals were treated once with MoDC or BMDC pulsed or not with P10 by subcutaneous route. The animals were euthanized 30 days after the DC treatment and the cytokines profile in the lungs was evaluated by CBA. Healthy (Sham) or infected and untreated mice (INFECT-only) were used as controls. Data shown are representative of three independent experiments, analyzed by one-way ANOVA followed by Tukey’s post-test, where ^*^p < 0.05, ^∗∗^*p* < 0.01, and ^∗∗∗^*p* < 0.001 in comparison to INFECT-only data set. IFN-γ, TNF-α, IL-2, IL-4, IL-10, and IL-12p70 levels did not show significant difference among the experimental groups. **(A–C)** Correspond respectively to cytokines, IL-16, IL-17A, MCP-1.

We also determined the levels of IL-12p70, IFN-γ, TNF-α, IL-2, MCP-1, IL-17A, IL-6, IL-10, and IL4 in the lungs of mice with different treatment regimens (Figure 5). A significant reduction of IL-6 and an increase in IL-17 production was observed in all groups that received BMDCs or MoDCs differentiated or not with P10 in comparison with infected control animals. Levels of MCP-1 were significantly increased only in the lungs of animals that were treated with MoDCs not differentiated with P10, albeit there was a trend toward increased amounts in the other DCs conditions. No significant differences were detected in IFN-γ, IL-12p70, TNF-α, IL-2, IL-4, and IL-10 levels (data not show).

**FIGURE 6:**
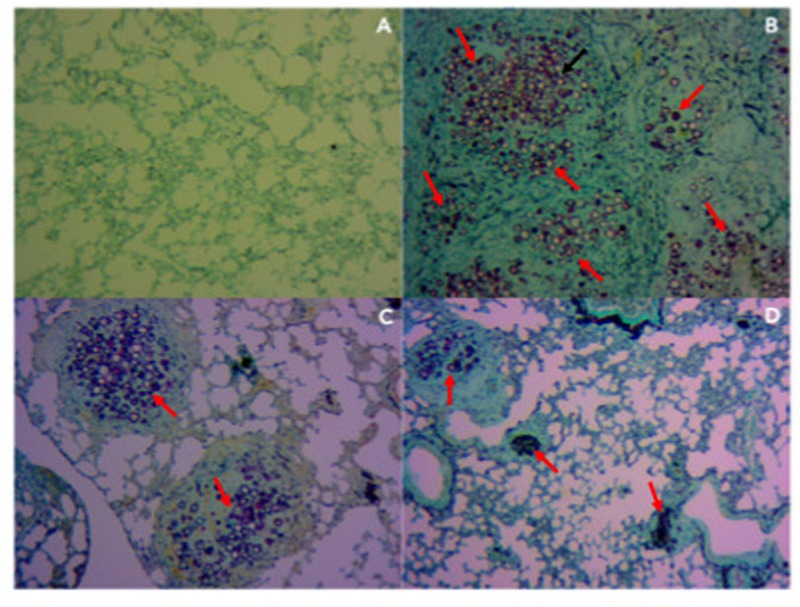
Therapy with BMDCs P10 and MoDC P10 reduces pulmonary fungal load in *P. brasiliensis* infected mice. Mice were infected with *P. brasiliensis*, and after 30 days the animals were treated once with MoDCs or BMDCs pulsed or not with P10 by subcutaneous route. The animals were euthanized 30 days after the DC treatment and the presence of yeasts in the lung tissue was evaluated by Gomori-Grocott stain. The red arrows show yeast in the pulmonary tissue. **(A)** Uninfected and untreated animals; **(B)** infected and untreated; **(C)** infected and treated with BMDC P10; **(D)** infected and treated with MoDC P10.

**FIGURE 7:**
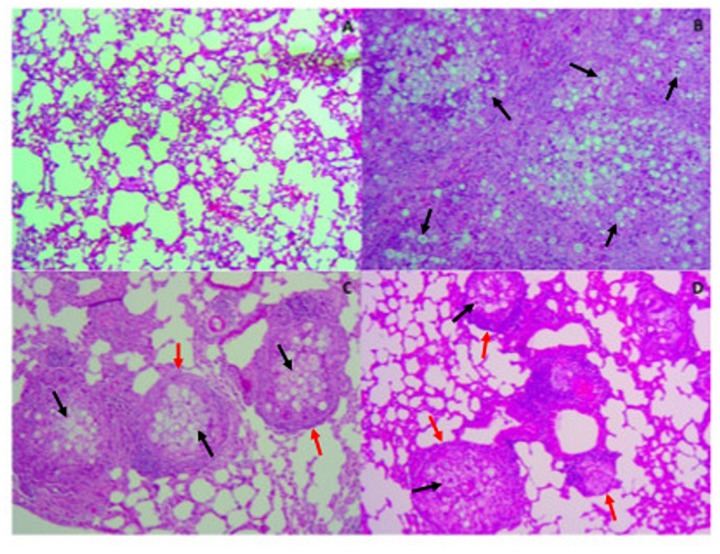
Therapy with P10-primed DCs reduces pulmonary damage in mice infected with *P. brasiliensis*. Mice were infected with *P. brasiliensis*, and after 30 days the animals were treated once with MoDC or BMDC pulsed or not with P10 by subcutaneous route. The animals were euthanized 30 days after the DC treatment and the lung was evaluated by Hematoxylin-eosin stain. The black and red arrows show yeast and granulomas in the pulmonary tissue, respectively. **(A)** Uninfected and untreated animals; **(B)** infected and untreated; **(C)** infected and treated with BMDC P10; **(D)** infected and treated with MoDC P10.

In order to evaluate tissue damage and fungal presence, Gomori-Grocott (Figure 6) and HE (Figure 7) staining of lung sections was performed. Figures 6A, 7A represent healthy mice lung tissue with normal architecture. Abundant fungal cells were diffusely present in the tissue of infected mice without treatment (Figure 6B), which also shows dense and diffuse inflammation (Figure 7B). Lung tissue from infected animals that received P10-differentiated BMDCs or MoDCs displayed clusters of fungal cells (Figures 6C,D, respectively) surrounded by granuloma-like inflammation (Figures 7C,D, respectively), but with larger areas of preserved parenchyma in comparison to non-treated, infected mice.

## Discussion

Several challenges complicate the effective resolution of PCM in human patients: the duration of therapy using current antifungal drugs lasts from months to years, yet this approach carries significant risks for severe side effects and it does not prevent unacceptable numbers of relapses (Shikanai-Yasuda et al., 2006). Moreover, the excessive inflammatory response in infected tissues contributes to PCM immunopathogenesis, and the subsequent tissue damage results in morbidity and may even result in death (Benard et al., 2012). Since PCM is not a compulsory notification disease, there are no precise data on its incidence. In Brazil, it is estimated that the annual incidence of PCM is between 0.71 and 3.7 cases per 100,000 inhabitants (Martinez, 2017). However, there are areas with higher numbers of cases, such as Rondônia State, reporting 9.4 cases per 100,000 inhabitants, with two cities having disease incidences close to 40 cases per 100,000 inhabitants (Vieira et al., 2014). Given the challenges of the antimicrobial therapy as well as the incidence and impact of PCM, our group has investigated different innovative, promising therapeutic approaches using peptide P10. P10 vaccination induces the development of a strong protective immune response that produces a significant reduction of fungal burden, tissue injury and fibrosis in a PCM murine model (Travassos et al., 2008; Taborda et al., 2015).

Previously, we demonstrated that therapy of murine PCM with BMDCs derived from healthy mice pulsed with P10 significantly reduced the fungal burden in lung tissue and stimulated a Th_1_-biased cytokine response (Magalhães et al., 2012). We then extended these findings by showing that BMDCs primed with P10 significantly reduced fungal burden in the lungs of immunocompromised mice in a disease model that simulates the most aggressive form of PCM (Silva et al., 2017). Therefore, we have pursued methods to optimize DC-based therapy for this disease. Since peripheral cells are significantly simpler to obtain than bone marrow from human patients, we have explored whether MoDC could modify PCM in our murine infection model.

At first, we observed that MoDCs are effectively generated from infected mice, albeit in lower percentages compared to BMDCs generation. The infection with Pb18 does not seem to affect the generation of both DCs types, since the percentages of CD11c^+^MHC-II^+^ cells recovered after differentiation are similar (data not shown). Stimulation with P10 induced both DCs activation, increasing their expression of MHC-II and CD83 maturation markers; the peptide was able to upregulate the CD80 expression only in CD8^+^ BMDCs. As a result, P10-primed BMDCs and MoDCs induced an effective increase in the proliferation of both CD8^+^ T and CD4^+^ T lymphocytes. Importantly, although P10 alone does not have the ability to stimulate proliferation of splenocytes, co-culture of splenocytes with BMDCs and MoDCs pulsed or not with P10 resulted in significant proliferation. These results emphasize the efficiency of DCs in P10 presentation and subsequent activation of sensitized or naive T cells (Steinman and Witmer, 1978). Furthermore, our data also indicates the presence of P10-reactive lymphocytes in the splenocytes of mice infected with *P. brasiliensis*.

In our results, the production of Th_1_/Th_2_/Th_17_ key cytokines in the co-culture supernatants was generally increased, with exception of IL-2, which was found in lower levels during splenocyte co-incubation with MoDCs primed or not. With the exception of IL-12p70, IFN-y, and IL-17, P10 stimulation significantly decreased the production of all other cytokines in comparison to unprimed DCs. Perhaps, overstimulation of those cytokine production contributes to lymphocyte anergy during incubation with unprimed DCs (observed by lack of lymphoproliferation), whereas P10 stimulation favor a balanced cytokine production that culminates with lymphocyte proliferation. The production of IL-12p70 stimulates IFN-γ generation, which is observed in the co-culture supernatants. Here, P10-primed DCs seem to induce higher expression of both cytokines, although differences were not statistically significant. This is of important note, considering that an effective cell-mediated immune response, with the presence of IL-12 and IFN-γ cytokines, is essential to promote PCM resolution (Travassos and Taborda, 2012). The decrease in the amount of IL-4 observed in the co-cultures with P10-primed DCs may be directly related to the ability of P10 to stimulate IL-12 production (Taborda et al., 1998), meanwhile the increase in IL-12 production causes suppression of IL-4 synthesis (Heufler et al., 1996).

After characterizing the phenotypes of MoDCs and BMDCs after differentiation and stimulation with P10, and analyzing their effects in lymphocyte response, we evaluated the therapeutic abilities during murine PCM. Our murine model is representative of what would likely be encountered in a clinical set, which is a human patient presenting an active PCM. Therefore, we accessed the ability of MoDCs and BMDCs, primed or not with P10, to reduce fungal burden and modify cytokine responses, as well as their impact in lung tissue architecture in infected mice. Since BMDCs and MoDCs are similar to the DCs found in lymphoid organs (Brasel et al., 2000), after subcutaneous administration, we expected that significant numbers of DCs would migrate to the draining lymph nodes within 24 h (Austyn, 2000; MartIn-Fontecha et al., 2003.) and thereafter participate in the immunological responses against *P. brasiliensis.* The administration of BMDCs or MoDCs resulted in a significant reduction in lung fungal burden, with the greatest reduction achieved when the BMDCs or MoDCs were pulsed with P10. These results were supported by histological analysis that revealed that therapy with P10-primed DCs significantly reduced the number of detectable yeast cells and greatly recovered lung parenchyma. The therapy using DCs reduced the presence of pro-inflammatory IL-6 to basal levels, while this cytokine remained increased in infected and non-treated mice. On the other hand, increased production of IL-17A in the lung tissue of DCs-treated mice was found, indicating the establishment of a T_reg_–biased immune response that correlated with reduced fungal burden.

In summary, our results indicate that, similarly, to BMDCs, P10-primed MoDCs are immunologically competent to protect the host against *P. brasiliensis* active infection during murine PCM. Hence, P10-primed MoDCs may be a significant advance for the treatment of complicated PCM. These findings support the use of autologous transplant of P10-primed MoDCs derived from peripheral blood cells of human patients to enhance host immune response and improve PCM outcome. Additionally, given the requirements for months to years of antifungal therapy using current regimens, P10-primed MoDCs may be useful in reducing the duration of drug treatment and possibly the incidence of relapse. Further investigation will determine if P10-primed MoDCs obtained from human patients, will also show the activation phenotypes and abilities to stimulate lymphocyte response as seen herein.

## Data Availability

The raw data supporting the conclusions of this manuscript will be made available by the authors, without undue reservation, to any qualified researcher.

## Ethics Statement

All procedures were performed according to the guidelines of National Council of Ethics with Animals (CONCEA) and the protocols were approved by the Ethical Committee for Animal Use from School of Medicine at University of São Paulo (certificate 189/14).

## Author Contributions

LS performed the experiments, analyzed the data, and wrote the manuscript. CLT, LD, and AS performed the experiments. AS, JN, LT, and CPT revised the manuscript. CPT designed the experiments, and wrote and revised the manuscript.

## Conflict of Interest Statement

The authors declare that the research was conducted in the absence of any commercial or financial relationships that could be construed as a potential conflict of interest.

## Supplementary Material

FIGURE S1 Analysis strategy used for phenotypic and activation profile of Bone Marrow-derived Dendritic Cells (BMDC). The cells were separated by granularity (SSC) and size (FSC), by viability, and then the live cells (live/dead –) were classified as BMDC based on double positivity for CD11c and MHC-II. BMDC were separated into two subpopulations, CD8^+^ and CD8^–^, and analyzed for their activation profile accordingly with the expression of CD80, CD86, and MHC-II. All gates were sat up with FMO (Fluorescence-Minus-One) controls.

FIGURE S2 Analysis strategy used for phenotypic and activation profile of Monocytes-derived Dendritic Cells (MoDC). The cells were separated by granularity (SSC) and size (FSC) and by viability. Then, the live cells double negative for CD115 and Ly6c were identified as monocyte-derived dendritic cells (MoDC) based on double positivity for CD11c and MHC-II. MoDCs were separated into two subtypes, CD8+ and CD8-, and were analyzed for their activation profile accordingly with the expression of CD80, CD86, and MHC-II. All gates were sat up with FMOs controls.

FIGURE S3 Analysis strategy used for the proliferation of T lymphocytes CD4^+^ and CD8^+^. Cells were selected by granularity (SSC) and size (FSC), by viability using the live/dead stain, and then identified as T lymphocytes based on the presence of the CD3 molecule on its surface. After that, the lymphocytes were classified as CD4^+^ and CD8^+^, and in each population the proliferation was analyzed with the CFSE stain. All gates were sat up with FMOs controls.
