# Supplementary figures and images for: Experimental Therapy of Paracoccidioidomycosis Using P10-Primed Monocyte-Derived Dendritic Cells Isolated From Infected Mice
Source: Front Microbiol. 2019 Jul 31;10:1727. doi: 10.3389/fmicb.2019.01727 (PMC6685297; doi:10.3389/fmicb.2019.01727)

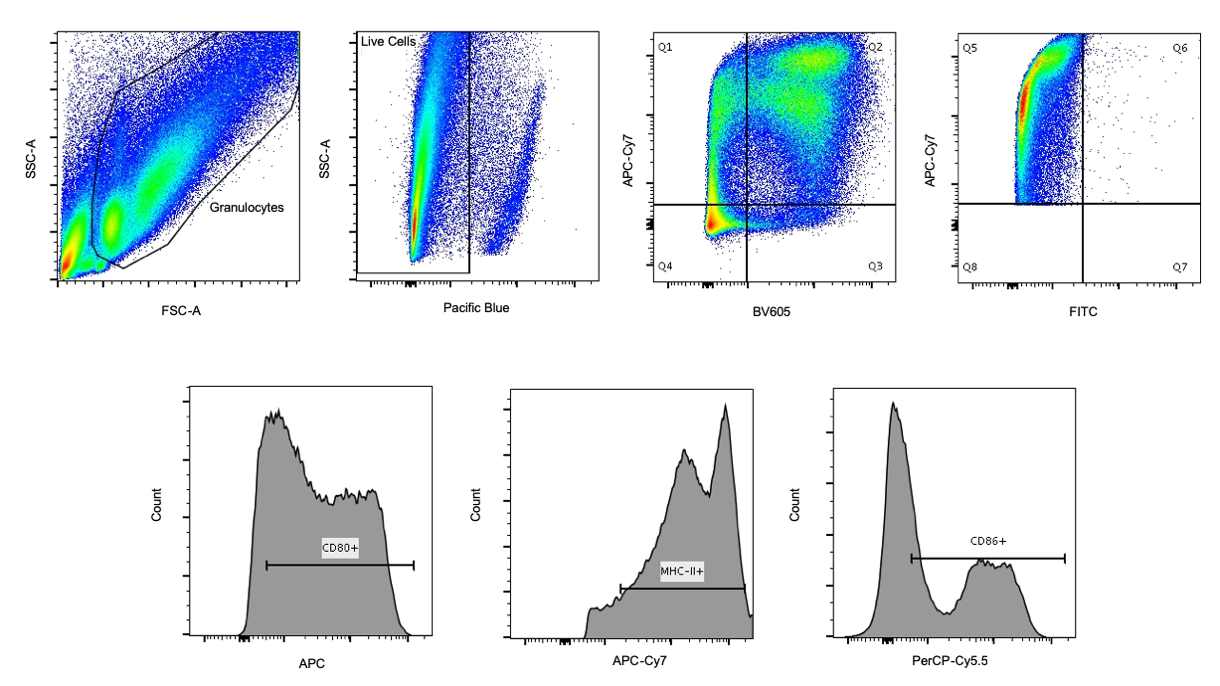

Supplement: FIGURE S1 — Analysis strategy used for phenotypic and activation profile of Bone Marrow-derived Dendritic Cells (BMDC). The cells were separated by granularity (SSC) and size (FSC), by viability, and then the live cells (live/dead –) were classified as BMDC based on double positivity for CD11c and MHC-II. BMDC were separated into two subpopulations, CD8+ and CD8–, and analyzed for their activation profile accordingly with the expression of CD80, CD86, and MHC-II. All gates were sat up with FMO (Fluorescence-Minus-One) controls. [file Image_1.TIF]

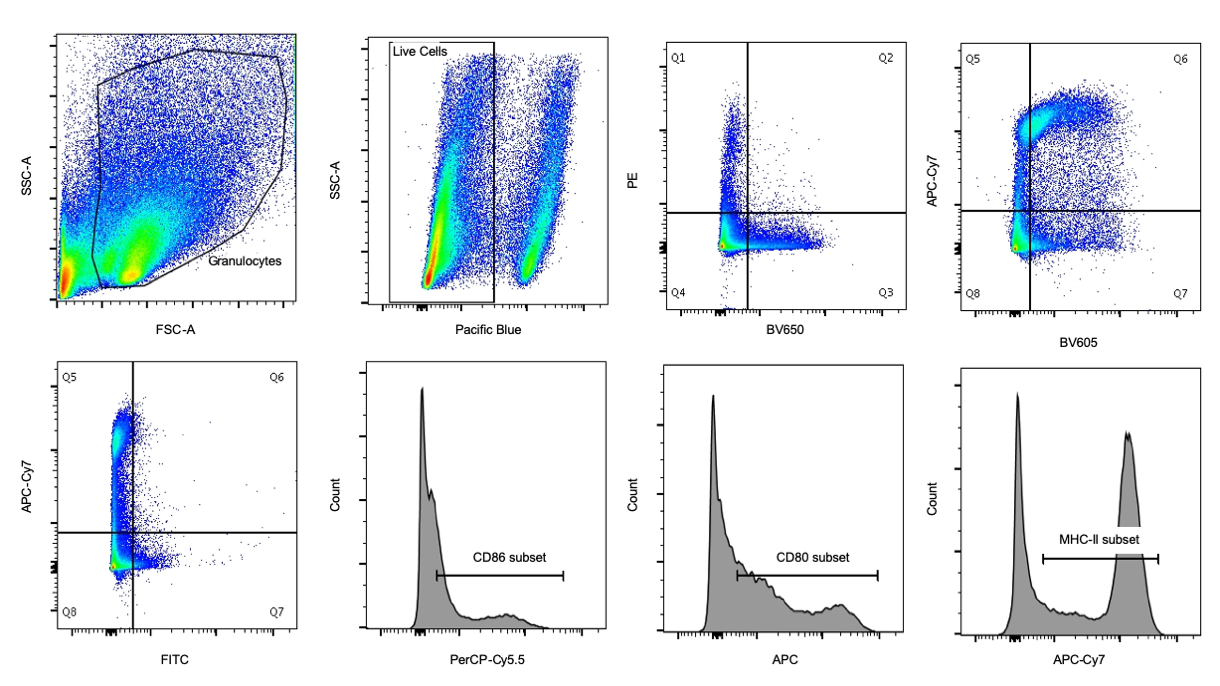

Supplement: FIGURE S2 — Analysis strategy used for phenotypic and activation profile of Monocytes-derived Dendritic Cells (MoDC). The cells were separated by granularity (SSC) and size (FSC) and by viability. Then, the live cells double negative for CD115 and Ly6c were identified as monocyte-derived dendritic cells (MoDC) based on double positivity for CD11c and MHC-II. MoDCs were separated into two subtypes, CD8+ and CD8-, and were analyzed for their activation profile accordingly with the expression of CD80, CD86, and MHC-II. All gates were sat up with FMOs controls. [file Image_2.TIF]

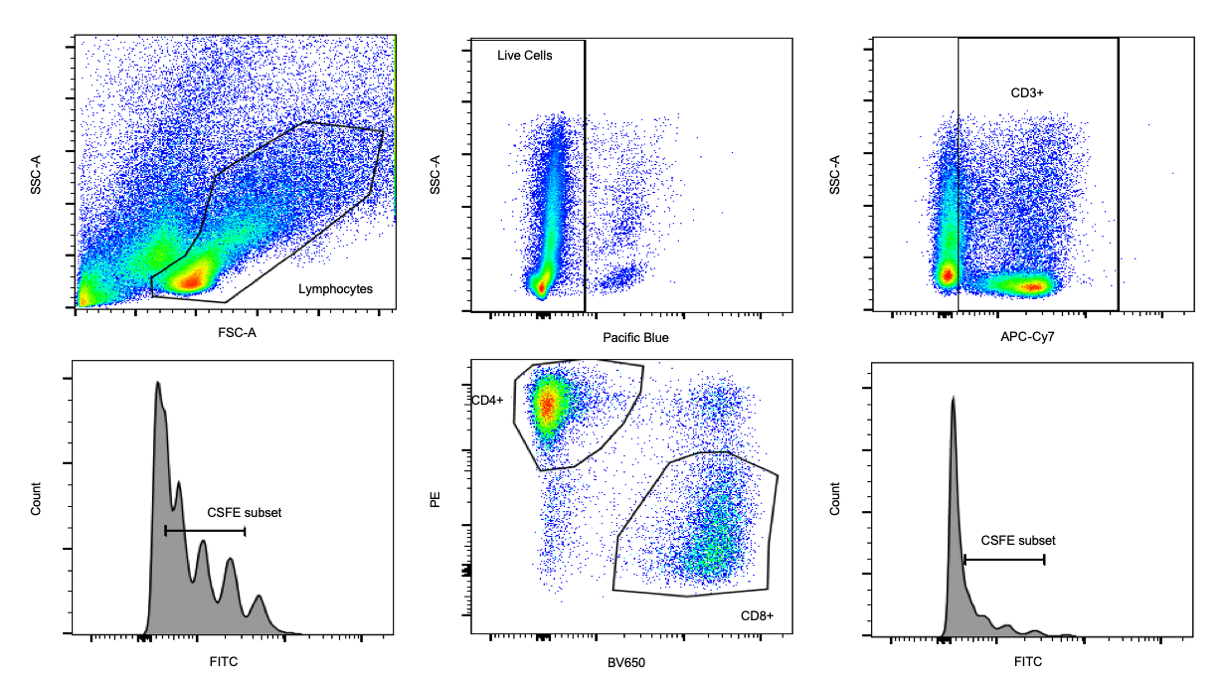

Supplement: FIGURE S3 — Analysis strategy used for the proliferation of T lymphocytes CD4+ and CD8+. Cells were selected by granularity (SSC) and size (FSC), by viability using the live/dead stain, and then identified as T lymphocytes based on the presence of the CD3 molecule on its surface. After that, the lymphocytes were classified as CD4+ and CD8+, and in each population the proliferation was analyzed with the CFSE stain. All gates were sat up with FMOs controls. [file Image_3.TIF]
